# A Lucrative Investment: Controlling Lead Paint Yields Big Dividends

**DOI:** 10.1289/ehp.117-a311a

**Published:** 2009-07

**Authors:** Tina Adler

**Affiliations:** **Tina Adler** first wrote for *EHP* about the Clinton–Gore environmental agenda in 1993. She is a member of the National Association of Science Writers and the American Society of Journalists and Authors

Fewer children today have the high blood lead levels seen in kids a few decades ago, when gasoline and paint still came fully leaded. But a surprising number of children still have blood lead levels that may place them at risk for a variety of cognitive, emotional, and behavioral problems. The good news is that boosting current efforts to protect U.S. children from one major source of lead—the house paint used prior to a 1978 ban, which still appears in many homes—may pay for itself many times over **[*EHP*** **117:1162–1167; Gould]**.

According to National Health and Nutritional Examination Survey data from 2003 to 2006, an estimated 25% of the 28 million U.S. children aged 6 years and younger have blood lead levels between 2 and 10 μg/dL, a range in which persistent cognitive damage is known to occur. Another 200,000 children are estimated to have levels over 10 μg/dL.

Using data from published studies, the author performed a cost–benefit analysis of the effects of controlling children’s exposure to lead paint. She calculated that controlling lead paint in the approximately 1 million worst-case housing units would cost between $1.2 billion and $11 billion, depending on many factors including local costs of lead abatement. But the benefits to be derived from controlling lead hazards could range from $181 billion to $269 billion. For example, abatement could save $11–53 billion in immediate medical treatment and $30–146 million in special education costs. Reducing the incidence of attention deficit/hyperactivity disorder (ADHD) related to lead paint exposure might save $267 million; and because both ADHD and lead exposure have been associated with criminal behavior, crime-related costs could shrink by $1.7 billion with efforts to eliminate and contain lead-laden paint.

The author concludes that every dollar spent to limit U.S. children’s exposure to lead paint—such as through paint stripping, replacement, and covering with a special encapsulant coating—could net $17–221. By comparison, vaccination against the most common childhood diseases is estimated to save $5.30–16.50 for every dollar spent on immunizations.

The author noted that the cost savings from better lead mitigation could be even higher than estimated in the current study. For one thing, the calculations of potential benefit pertain only to children under age 6. Yet getting rid of lead paint would benefit other segments of the population as well. Also, the analysis excluded many potential costs of lead exposure, including future health care expenses and the indirect costs of criminal activity.

U.S. public health and housing policies have been slow to address the lingering problems related to lead paint, the author asserts. Given the huge projected savings and income in terms of health care, crime prevention, education, lifetime earnings, and tax revenues, she writes, the time for proactive and universal lead control has never been better.

## Figures and Tables

**Figure:**
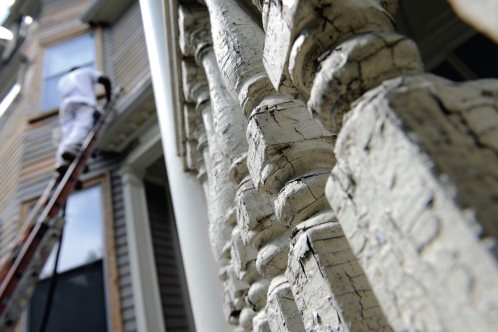
It can cost a homeowner more than $200,000 to clean up lead contamination after uncontrolled power sanding, compared with about $1,200 to incorporate lead-safe work practices into repainting.

